# Size-Dependent Mechanical Properties of Amorphous SiO_2_ Nanowires: A Molecular Dynamics Study

**DOI:** 10.3390/ma13225110

**Published:** 2020-11-12

**Authors:** Kun Sun, Juan Chen, Bingjie Wu, Liubing Wang, Liang Fang

**Affiliations:** 1 State Key Laboratory for Mechanical Behavior of Materials, Xi’an Jiaotong University, Xi’an 710049, China; 2 School of Materials Science and Engineering, Taiyuan University of Science and Technology, Taiyuan 030024, China; juanchcumt@126.com; 3 Nuclear Power Institute of China, Chengdu 610014, China; wubingjie91923@163.com (B.W.); wangliubing@163.com (L.W.); 4 School of Mechanical & Electrical Engineering, Xiamen University Tan Kah Kee College, Zhangzhou 363105, China

**Keywords:** uniaxial tension, amorphous SiO_2_ nanowires, mechanical properties, plastic deformation, molecular dynamics simulation

## Abstract

Uniaxial tension tests were performed for amorphous SiO_2_ nanowires using molecular dynamics simulation to probe the size effect on the mechanical properties and plastic deformation by varying the length of nanowires. The simulation results showed that the Young’s modulus of SiO_2_ nanowires increased with the decrease of nanowires length due to its higher surface stress. The corresponding deformation of SiO_2_ nanowires during tension exhibited two periods: atomic arrangement at small strain and plastic deformation at large strain. During the atomic arrangement period, the percentage variations of atom number of 2-coordinated silicon and 3-coordinated silicon (PCN2 and PCN3) decreased, while the percentage variations of atom number of 4-coordinated silicon, 5-coordinated silicon (PCN4 and PCN5) and the Si–O bond number (PCB) rose slightly with increasing strain, as the strain was less than 22%. The situation reversed at the plastic deformation period, owing to the numerous breakage of Si–O bonds as the strain grew beyond 22%. The size effect of nanowires radius was considered, finding that the Young’s modulus and fracture stress were higher for the larger nanowire because of fewer dangling bonds and coordinate defeats in the surface area. The elastic deformation occurred at a small strain for the larger nanowire, followed by the massive plastic deformation during tension. A brittle mechanism covers the fracture characteristics, irrespective of the nanowire size.

## 1. Introduction

Amorphous SiO_2_ is an inorganic material that has various applications in many nanotechnology areas, such as nanoelectronics, microfluidics and sensors [1]. Many attempts have been made to investigate the structure and mechanical properties of amorphous SiO_2_. Structural transformation from tetrahedral to octahedral transformation of densified SiO_2_ was studied [2,3], and a comprehensive relationship between structure, physical and electronic properties was provided by Wu [4], suggested that the compression mechanism is linked to the appearance of 5- and 6-coordinated silicon. The broken Si–O bond linked to 4-coordinated silicon is attributed to the plastic flow through its capacity to change the number of silicon atoms within a Si–O–Si–O ring [5]. The distribution of the Si–O ring affects the plastic deformation and fracture behaviors of amorphous SiO_2_ by varying the quenching rate [6]. Experimental observations and theoretical analysis revealed that the nanocavities existing ahead of or around the pre-crack/crack coalesce with kinks to form crack nanocolumns, the newly formed crack nanocolumns, finally develop into a crack [7,8,9].

Nanowires exhibited a size-dependent effect under tension and bending tests, i.e., the Young’s modulus changes as the nanowire diameter shrinks down to the nanoscale. The experimental observation indicated that the Young’s modulus of Si and GaN nanowires decreases [10,11] while it increases for ZnO nanowires with the decreasing of the diameter [12]. The decreasing modulus with decreasing size in <111> Si is found to be more apparent than that obtained for Si nanowires along <110> and <100> [13]. The SiO_2_ nanowires prepared by different methods exhibit different values of bending modulus, 68 GPa [14], 76 GPa [15,16] and 47 GPa for the sample with 100 nm diameter [17]. It suggested [16] that elastic weakening occurs to the nanowires with intermediate diameters (43 to 98 nm), while an increased modulus of 89–101 GPa turns out for nanowires with diameters of 3.7–6.0 nm measured by molecular dynamics (MD) simulation using the pairwise potential developed by van Beest, Kramer and van Santen (BKS) [18]. The MD simulations with reactive force field ReaxFF were conducted and observed a tension modulus of 19–30 GPa for SiO_2_ nanowires; the tension modulus decreases as the size decreases [19]. The influence of preparation methods (casting, cutting and cutting–straining) on the elastic response of SiO_2_ nanowires was investigated by Yuan et al. [20] and an eigenstress model was developed to explain the size-dependent modulus. The reason for the size effect is still in discussion; possible reasonable reasons include (i) surface effect (surface stress and surface elasticity), (ii) nonlinear elastic response of the nanowires core [11,21], (iii) presence of an oxide and surface defects at the surface of nanowires and (iv) different potentials used in MD simulations. The influence of surface stress on the equilibrium lattice spacing and biaxial Young’s modulus is explored [22,23], and the surface phase and edge phase result in the size dependence of modulus through theoretically dividing the nanowires into different phase (hypothetical nanowire phase, surface phase and edge phase), the surface and edge effects are more significant with the decreasing diameter [24]. The native oxide and defects can reduce the modulus because of its lower modulus [25,26]. Although much studies have been dedicated, a thorough understanding of amorphous SiO_2_ is still absent, especially at the nanoscale.

This work aims to conduct uniaxial tension tests for amorphous SiO_2_ nanowires with a constant diameter but various lengths using molecular dynamics simulation, shedding light on the effect of length on the mechanical properties and plastic deformation behaviors of amorphous SiO_2_ nanowires at the nanoscale.

## 2. Modeling and Methods

To test the length effect of cylindrical nanowire on the mechanical properties and plastic deformation behaviors, a uniaxial tension test was performed using the large-scale atomic/molecular massively parallel simulator (LAMMPS) [27]. The cubic amorphous SiO_2_ was first prepared by quenching melted cristobalite [28] and then carved into cylindrical nanowires with the same diameter (6 nm) but different lengths. The nanowires with various lengths of 10, 20, 30, 40, 50 and 100 nm contained 18,970, 36,950, 55,048, 73,069, 91,094 and 180,980 atoms, respectively. The three-body Tersoff potential was extended to the Si–O system based on the ab initio calculations by Munetoh [29] and has been successfully used to describe interactions of amorphous SiO_2_. Thus, in this work, the extended Tersoff potential was employed to depict the interatomic interactions between Si–Si, O–O and Si–O atoms.

**Figure 1:**
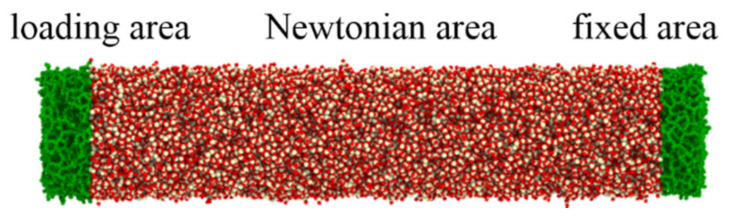
Schematic of the molecular dynamics (MD) simulation of uniaxial tension for amorphous SiO_2_ nanowire.

The atoms within cylindrical nanowires were divided into three areas: the fixed area, the loading area and the Newtonian area, as shown in Figure 1. The fixed area, containing 2980 atoms, was positioned at the bottom of the nanowire and was frozen to stabilize the nanowire during tension. The loading area, which was designed as a rigid body at the other side of the nanowire, could transmit stress from the loading area to the Newtonian area. The atoms within the Newtonian area were sandwiched between the fixed area and the loading area. These atoms could freely move according to Newton’s second law. Before loading, the nanowire was first relaxed to local energy minimum by the steepest descent algorithm. It was then relaxed using the canonical (NVT) ensemble with the Nose–Hoover thermostat for 60 ps to reduce the global potential energy of the nanowire. After stabilization of the nanowire, the uniaxial tension was performed through displacing the atoms within the loading area at a constant strain rate of 2.5 × 10^7^ with a time step of 0.5 fs. The tension system maintained a constant temperature of 300 K by using the NVT ensemble during the whole tension process. Non-periodic boundary conditions were applied in three axes.

## 3. Results and Discussion

### 3.1. Mechanical Properties

**Figure 2:**
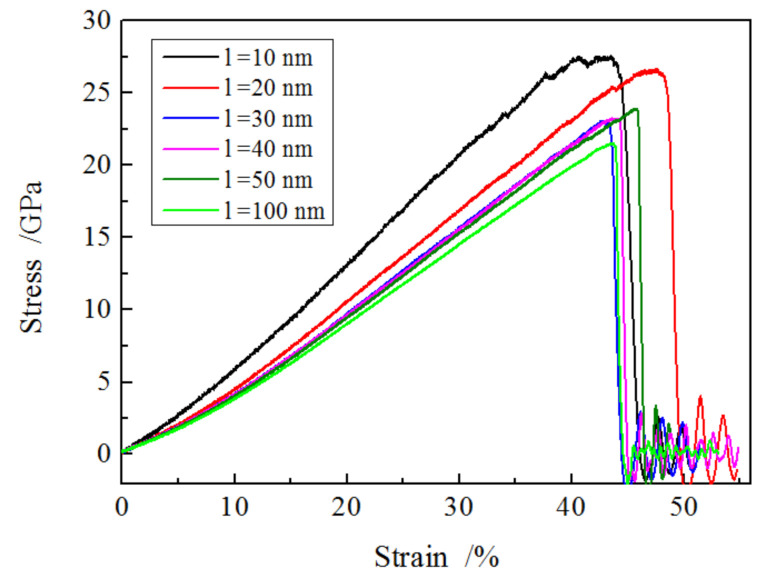
The relationship between stress and strain during uniaxial tension for nanowires with various lengths.

A series of uniaxial tension tests was conducted for different cylindrical nanowires with the same diameter (6 nm) but various lengths (10, 20, 30, 40, 50 and 100 nm); the detailed simulation process is given in the above section. The applied stress and strain of the nanowires during tension are monitored, and the relationship between the two was obtained and summarized in Figure 2; it showed that the stress increased steadily with the growing strain, then declined quickly after reaching its fracture strength, meaning a brittle mechanism occurred in the SiO_2_ nanowires. It was found that there was a decrease in applied stress with increasing nanowire length at the same strain prior to fracture, e.g., 17 GPa and 12 GPa for the length of 10 nm and 100 nm nanowires at the strain of 25%, respectively. The fracture strength of the nanowires grew with decreasing length, which was in accordance with Han’s results [30].

At the initial period of the stress and strain curves, the stress was proportional to the strain, which suggests that the elastic deformation took place to amorphous SiO_2_ nanowires, and the linear section of the curve gives the Young’s modulus, which was determined through the ratio of applied stress to strain. The calculated Young’s modulus shown in Table 1 shows that the modulus increased with the decreasing length of nanowires, exhibiting a strong size effect, i.e., the shorter, the stronger. The determined values of Young’s modulus were consistent with those obtained using reactive force field Reaxff (19–30 GPa) [19] but higher than those determined in BKS potential (89–101 GPa) [16].

### 3.2. Plastic Deformation

**Figure 3:**
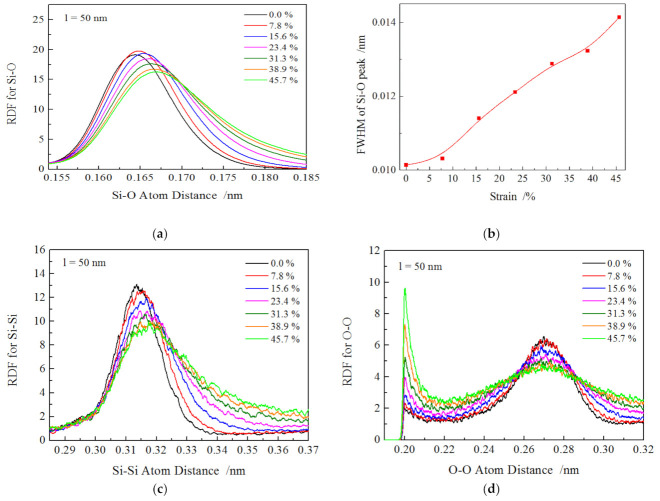
Radial distribution function (RDF) of three atom pairs and the full width at half maximum of Si–O peak (FWHM) for amorphous SiO_2_ nanowire with a length of 50 nm at different strains. (**a**) Si-O; (**b**) the full width at half maximum of Si–O peak (FWHM); (**c**) Si-Si; (**d**) O–O.

The amorphous SiO_2_ is composed of numerous randomly distributed ordered SiO_4_ tetrahedra. To investigate the plastic deformation characteristics of amorphous SiO_2_ during tension, the radial distribution function (RDF) of three atom pairs was obtained, as shown in Figure 3. As the tension proceeded, the Si–O peak shifted right, its height declines while its width (or the full width at half maximum) of Si–O peak (FWHM) in Figure 3b) increased as the strain grew, which implies that the average Si–O distance enlarged from equilibrium value (0.162 nm for the as-prepared nanowires, close to the values of experiment measurement [31]) to a larger value of 0.167 nm, as shown in Figure 3a. The Si–Si RDF exhibited a similar trend to that of Si–O RDF in Figure 3c. The RDF of O–O atom pair is shown in Figure 3d. It was found that the average O–O atomic distance was 0.265 nm at the strain of 0.0%, which was also in accordance with the experimental results [31]. Moreover, it is interesting to note that an extra peak appeared in the O–O RDF at the distance of about 0.2 nm; the extra peak expanded apparently while the main peak of O–O RDF decreased with the increasing strain. In addition, there was an extra small peak in O–O RDF at the strain of 0.0%, which differed from the result of amorphous SiO_2_ at the strain of 0.0% during loading [32,33,34,35], where no extra O–O peak appeared. This difference may have been caused by the high ratio of specific surface area to volume of cylindrical nanowires compared with amorphous SiO_2_ films and its bulk counterparts.

**Figure 4:**
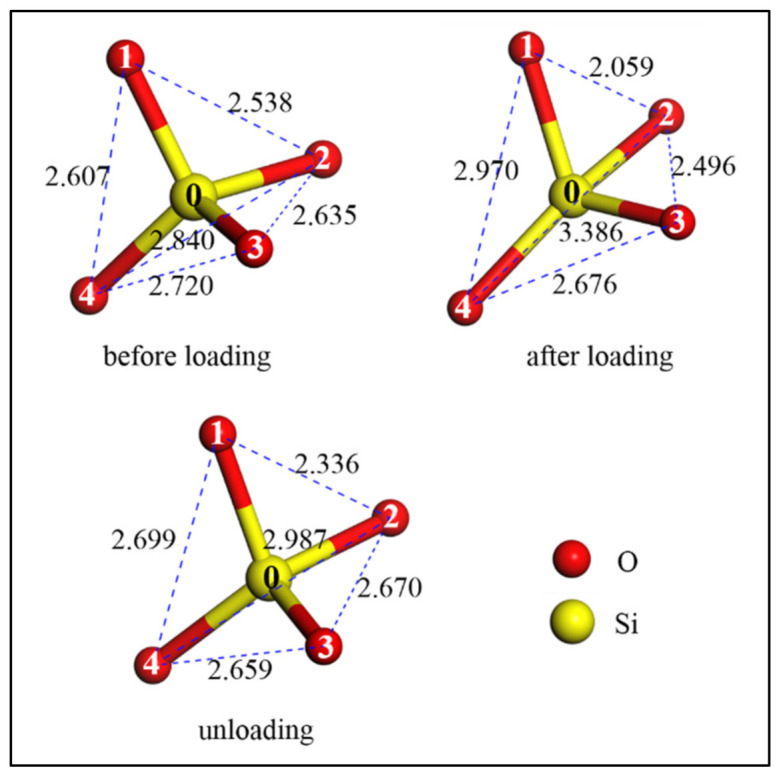
Changes of SiO_4_ tetrahedron during tension (units: Å).

The reason for the appearance of an extra O–O peak can be explained by Figure 4. It shows that in the as-prepared amorphous SiO_2_ cylindrical nanowire (before loading), the SiO_4_ tetrahedron within it was almost short-range ordered, and O–O atomic distances were 0.2538, 0.2635, 0.2720, 0.2840 and 0.2607 nm between oxygen atoms O_1_–O_2_, O_2_–O_3_, O_3_–O_4_, O_2_–O_4_ and O_1_–O_4_, respectively. As the tension strain increased to 45.7%, the original ordered SiO_4_ tetrahedron became stretched along the loading direction. The corresponding O–O atomic distances were hence changed to 0.2059, 0.2496, 0.2676, 0.3386 and 0.2970 nm, respectively. The massive reduction of O–O atomic distance within SiO_2_ (e.g., O_1_–O_2_ atoms) during tension leads to the increasing of the extra peak in the O–O RDF at a distance of 0.2 nm. After unloading, elastic recovery occurred to these O–O atoms and the corresponding distances changed to 0.2336, 0.2670, 0.2659, 0.2987 and 0.2699 nm, respectively. Conclusively, the original short-range ordered SiO_4_ tetrahedra within the as-prepared SiO_2_ became disordered during tension, and these disordered SiO_4_ tetrahedra could not recover to their original state even as the stress was released, indicating the plastic deformation indeed occur to amorphous SiO_2_ nanowire by the deformation of numerous SiO_4_ tetrahedra. The changes of RDF for amorphous SiO_2_ nanowire during tension were similar to those for previous indentation tests, where the SiO_4_ tetrahedron was flatted due to indentation [32,33,34,35].

**Figure 5:**
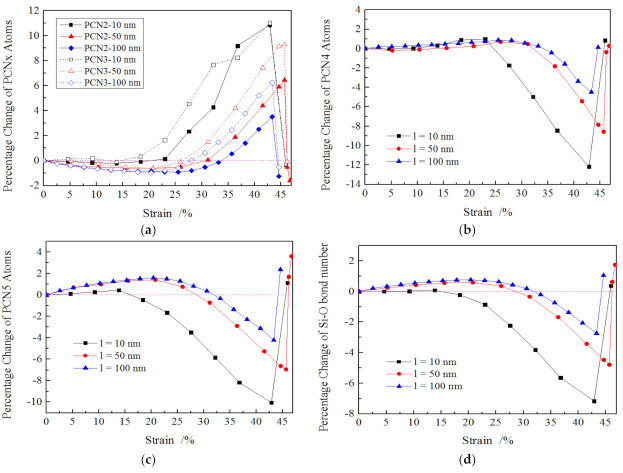
Percentage change of atom number of x-coordinated silicon (PCNx) and number of Si–O bonds (PCB) for nanowires with different lengths. (**a**) PCN2 and PCN3 (**b**) PCN4 (**c**) PCN5 (**d**) Si–O bond.

To further probe the plastic deformation characteristics of amorphous SiO_2_ nanowires, the number variations of coordinated silicon atoms within amorphous SiO_2_ were carefully analyzed in the form of percentage change of atoms number of x-coordinated silicon (PCNx) and percentage change of the number of Si–O bonds (PCB). The PCNx and PCB are described in Equations (1) and (2). The CNx denotes x oxygen atoms were bonded to one silicon atoms. The careful analysis of PCNx and PCB within a cutoff of 0.2 nm was calculated and plotted in Figure 5. It was found that as the strain increased from 0% to about 22%, the PCN2 and PCN3 decreased slightly depending on the length of the nanowires, as illustrated in Figure 5a. The longer the nanowire, the more PCN2 and PCN3 decreased. Subsequently, the PCN2 and PCN3 increased sharply as the strain rose until the fracture occurred to the nanowire. The values of PCN2 and PCN3 rose with the decreasing length of nanowires at the same strain, e.g., the values of PCN2 were 4%, 1% and 0.5% for 10, 50 and 100 nm long nanowires at the strain of 30%, respectively. The PCN4 increased slightly, and its values were a litter higher than 0.0% as the strain was less than 22%, then PCN4 decreased dramatically with the increasing strain. In addition, the values of PCN4 grew with increasing length of the nanowires until its fracture occurred, as shown in Figure 5b.

(1) $$PCNx=\frac{CNx\;atom\;number_{strain=6.0\;\%}-CNx\;atom\;number_{strain=0.0\;\%}}{CN4\;atom\;number_{strain=0.0\;\%}}\times 100\%$$

(2) $$PCB=\frac{bond\;number_{strain=6.0\;\%}-bond\;number_{strain=0.0\;\%}}{bond\;number_{strain=0.0\;\%}}\times 100\%$$

As the tension strain rose, the PCN5 increased first and had a maximum value at the strain of ~22%, then declined distinctly until the nanowires fracture. The values of PCN5 were higher for longer nanowire at the same strain, e.g., it was −4.6% for 10 nm-long nanowires and 0.4% for 100 nm long nanowire at the strain of 30%. The PCB value was a net result of the formation of Si–O bonds owing to atomic rearrangement (compression induced by surface stress) and breakage due to severe plastic deformation, namely the PCB was a synthesis result of PCN2, PCN3, PCN4 and PCN5, where the increase in PCN2 and PCN3 meant the decline in PCB, while the increase in PCN4 and PCN5 implied the increase in PCB. The variations of PCB during tension are shown in Figure 5d; it exhibits a variation tendency similar to that of PCN5, but with a relatively lower value.

According to the above analysis, we could conclude that plastic deformation during loading exhibits two periods: atomic rearrangement and fracture periods. At the atomic rearrangement period (the strain was less than 22%), there was a decrease in PCN2, PCN3 and an increase in PCN4 and PCN5 on the contrary, leading to the increased values in PCB. The effect of atomic rearrangement enhances for longer nanowire. As the strain increased beyond 22%, the values of PCN2 and PCN3 grew sharply, while PCN4 and PCN5 declined dramatically. This indicates that numerous Si–O bonds in the SiO_4_ and SiO_5_ structure of SiO_2_ nanowires broke under higher applied stress. When the increased Si–O bond number cannot compensate for the decreased Si–O bonds, the fracture of the nanowire tended to occur.

**Figure 6:**
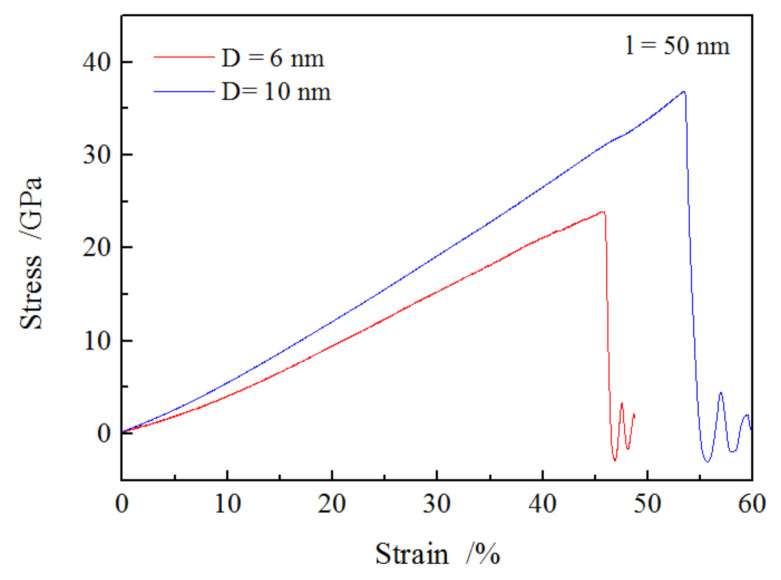
The tension curves of stress and strain for SiO_2_ nanowires with different diameters.

To more deeply investigate the effect of nanowire size on the tension properties and plastic deformation, an additional tension test for SiO_2_ nanowire with a diameter of 10 nm and a length of 50 nm was performed. The relationship between stress and strain is illustrated in Figure 6; the stress–strain curve of SiO_2_ nanowire with 6 nm-diameter is included for comparison. It showed that the applied stress for the SiO_2_ nanowire with a diameter of 10 nm was significantly higher than that of 6 nm at the same strain, as well as the fracture stress and the corresponding strain. The determined modulus for 10 nm-long nanowires was 44.7 GPa, which was 46% higher than that of 6 nm (30.6 GPa). This was consistent with the modulus obtained by experiments [11,36] and molecular dynamics simulation [19]. Although the surface effect could influence the elastic property due to the high ratio of specific surface area to volume in small nanowires, which could enhance the stiffness of nanowires [20], the much more dangling bonds and coordination defects (Si and O atoms with less coordination number) in surface area of the small nanowire, coupled with fewer internal atoms in small nanowires reduce the Young’s modulus [37].

**Figure 7:**
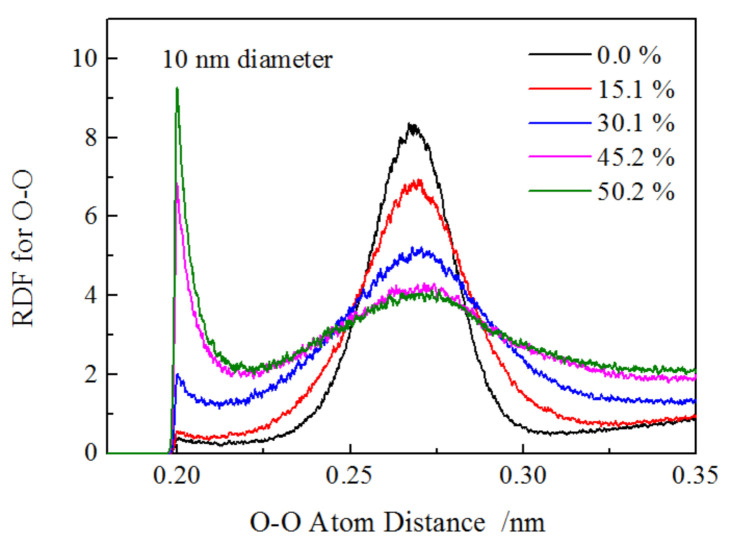
O–O RDF for 10 nm-diameter nanowires.

The O–O RDF for larger nanowire (10 nm-diameter) was investigated as well and is illustrated in Figure 7; it was found that the variation trend of the main peak and extra peak resembled that of the small nanowire. The main difference was that the height of extra peak for the larger nanowire was 0.4, and the ratio of extra peak height to main peak height was 0.048, which was much lower that of 6 nm-diameter nanowires (2 for extra peak height, 0.303 for the ratio) at the strain of 0.0%. From these results, we could conclude that the shorter and smaller nanowire exhibited a stronger size effect, causing the severe atomic arrangement occurring in the small nanowire due to its higher surface stress.

**Figure 8:**
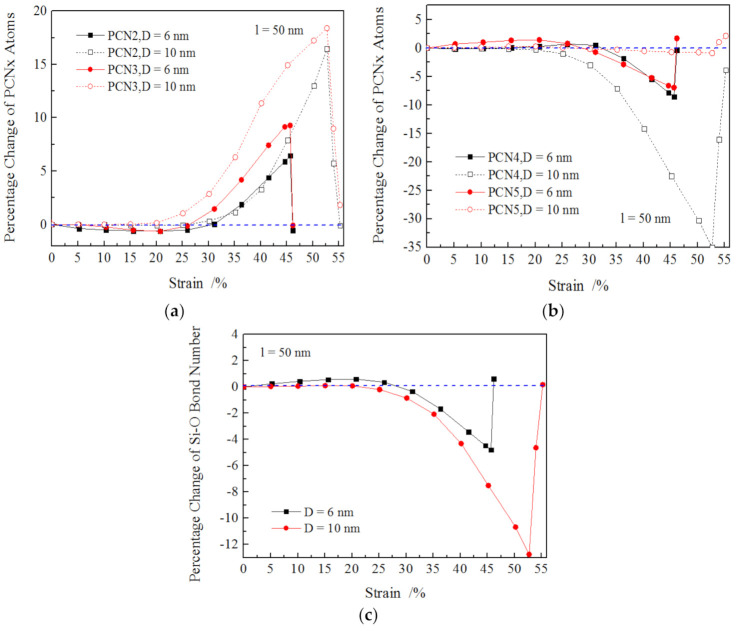
Percentage change of atom number of x-coordinated silicon (PCNx) and the number of Si–O bonds (PCB) for nanowires with different diameters. (**a**) PCN2 and PCN3, (**b**) PCN4 and PCN5, (**c**) Si–O bonds.

The changes of PCNx and PCB for nanowires with different diameters were obtained as shown in Figure 8, revealing that in a small strain range (less than about 20%), the values for PCNx (x = 2, 3, 4 and 5) and PCB of 10 nm-diameter nanowires kept an almost constant value of zero. The results imply that elastic deformation may have occurred to the larger SiO_2_ nanowire instead of the atomic rearrangement, which took place to the nanowires with a small diameter and was induced by the size effect (higher ratio specific surface area to volume). For the 10 nm-diameter nanowires, as the strain increase exceeded 20%, PCN2 and PCN3 increased sharply while PCN4, PCN5 and PCB declined dramatically until the fracture of SiO_2_ nanowire occurred. It was also found that the absolute values of PCNx (x = 2, 3, 4 and 5) and PCB for 10 nm-diameter nanowires were much higher than those of 6 nm at the same strain, implying that massive plastic deformation occurred to the 10 nm-long nanowires and much more Si–O bonds broke; thus the corresponding applied stress was significantly higher than the small nanowire. It could be inferred from the above results that the appearance of the extra O–O peak and the decrease in PCN2 and PCN3 for small nanowire at the small strain during tension resulted from atomic arrangement owing to the size effect. As the size decreased to the nano or atomic scale, the increasing surface-to-volume ratio lead to enhanced surface stress, which resulted in compression stress in the surface region and tension stress in the inner part of nanowires. This was verified by the appearance of an extra O–O peak for the as-prepared SiO_2_ nanowire after sufficient relaxation.

**Figure 9:**
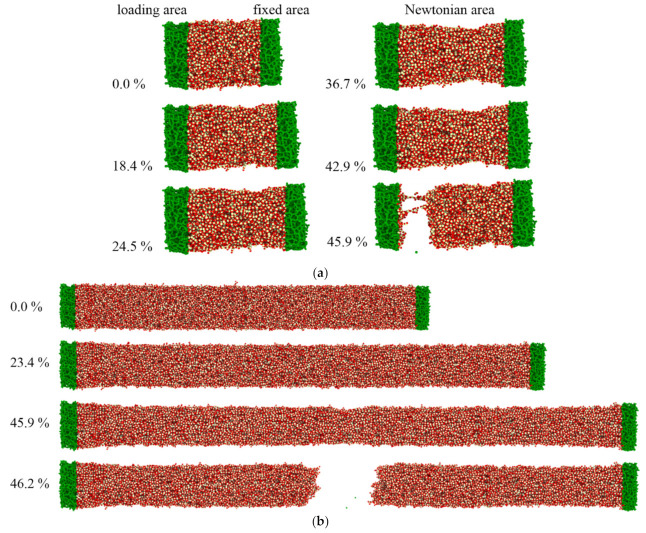
Atomic configuration of SiO_2_ nanowires during tension as a function of strain. (**a**) SiO_2_ nanowire with radius 6 nm and length of 10 nm and (**b**) SiO_2_ nanowire with radius 10 nm and length of 50 nm.

The atomic configuration of deformation evolution as a function of strain is illustrated in Figure 9; it was found that for SiO_2_ nanowire with a length of 10 nm within a small strain (~18.4%), the nanowire was stretched almost evenly. Then pronounced local plastic deformation occurred as the strain grows, finally the nanowire fractured at the strain of about 44%. For larger nanowire with a radius of 10 nm and length of 50 nm, the nanowire deformed homogeneously during whole tension until the fracture takes place. These results indicate that the plastic deformation characteristics for two nanowires were different and that brittle mechanism occurred to the nanowires inferred from its cross-section after fracture, regardless of its diameter. It also illustrates that the longer the nanowire was, the more uniform the deformation. The detailed plastic deformation mechanism has been published elsewhere [33].

## 4. Conclusions

Uniaxial tension tests were conducted for amorphous SiO_2_ nanowires with the same radius but different lengths using molecular dynamics simulations; the results are concluded as follows:

(1) The stress and strain curves revealed a strong size effect; the calculated Young’s modulus increased with the decreasing length of nanowires, as well as the fracture stress;
(2) The Si–O peak shifted towards the larger distance during tension; the height of the Si–O peak decreased while the width increased based on the RDF analysis with the increasing tension strain. An extra peak appears in O–O RDF due to the numerous deformation of SiO_4_ tetrahedra, and the peak enlarges during tension;
(3) The deformation of SiO_2_ nanowires with a radius of 6 nm exhibited two periods during tension: atomic arrangement and plastic deformation periods. The PCN2 and PCN3 decreased, while PCN4, PCN5 and PCB increased slightly owing to the higher surface stress at the atomic arrangement period. The PC2 and PCN3 grew sharply, while PCN4, PCN5 and PCB declined dramatically at plastic deformation periods before the fracture of nanowires occurred;
(4) There was an increase in Young’s modulus for larger nanowire due to fewer dangling bonds and coordinate defeats compared with small nanowires. The elastic deformation for larger nanowire dominated at small tension strain instead of the atomic arrangement that happened to the small nanowire.

## Figures and Tables

**Table 1:** The obtained Young’s modulus for nanowires with different length (l: nm).

Lengths/nm	10	20	30	40	50	100
Modulus/GPa	44.6	34.3	32.2	31.4	30.6	29.2
